# Plasma extracellular RNA profiles in healthy and cancer patients

**DOI:** 10.1038/srep19413

**Published:** 2016-01-20

**Authors:** Tiezheng Yuan, Xiaoyi Huang, Mark Woodcock, Meijun Du, Rachel Dittmar, Yuan Wang, Susan Tsai, Manish Kohli, Lisa Boardman, Tushar Patel, Liang Wang

**Affiliations:** 1 Department of Pathology and MCW Cancer Center, Medical College of Wisconsin, Milwaukee, WI 53226, USA; 2 Center for Biotherapy, the Second Affiliated Hospital of Harbin Medical University, Harbin, Postcode 150086, China; 3 Department of Internal Medicine, Medical College of Wisconsin, Milwaukee, WI 53226, USA; 4 Department of Surgery, Medical College of Wisconsin, Milwaukee, WI 53226, USA; 5 Department of Oncology, Mayo Clinic, Rochester, MN 55905, USA; 6 Departments of Transplantation and Cancer Biology, Mayo Clinic, Jacksonville, FL 32224, USA

## Abstract

Extracellular vesicles are selectively enriched in RNA that has potential as disease biomarkers. To systemically characterize circulating extracellular RNA (exRNA) profiles, we performed RNA sequencing analysis on plasma extracellular vesicles derived from 50 healthy individuals and 142 cancer patients. Of ~12.6 million raw reads for each individual, the number of mappable reads aligned to RNA references was ~5.4 million including miRNAs (~40.4%), piwiRNAs (~40.0%), pseudo-genes (~3.7%), lncRNAs (~2.4%), tRNAs (~2.1%), and mRNAs (~2.1%). By expression stability testing, we identified a set of miRNAs showing relatively consistent expression, which may serve as reference control for exRNA quantification. By performing multivariate analysis of covariance, we identified significant associations of these exRNAs with age, sex and different types of cancers. In particular, down-regulation of miR-125a-5p and miR-1343-3p showed an association with all cancer types tested (false discovery rate <0.05). We developed multivariate statistical models to predict cancer status with an area under the curve from 0.68 to 0.92 depending cancer type and staging. This is the largest RNA-seq study to date for profiling exRNA species, which has not only provided a baseline reference profile for circulating exRNA, but also revealed a set of RNA candidates for reference controls and disease biomarkers.

Extracellular vesicles such as exosomes and microvesicles are present in all types of biological fluids (e.g. blood)^1^. Exosomes are cell-derived membrane vesicles (30–100 nm) consisting of a lipid bilayer membrane surrounding a small cytosol and are believed to be involved in various biological functions including angiogenesis, cell proliferation, tumor cell invasion and metastasis, immune response and antigen presentation through intercellular transfer of their protein or RNA content^2^,^3^. Due to their important roles in intercellular communication, extracellular vesicles have been evaluated as carriers to deliver therapeutic agents across biological membranes^4^,^5^. In addition, extracellular vesicle contents often demonstrate dynamic changes to reflect their origin and disease status. Therefore, extracellular vesicles have a great potential as important source material for biomarker discovery^6^,^7^.

Circulating extracellular vesicles in blood have been described as treasure chests for cancer biomarkers. It has been reported that extracellular vesicles contains a variety of RNA species, the majority being small non-coding RNAs. Because of their relative stability within vesicles, these extracellular RNAs (exRNAs) have been examined as potential biomarkers for various cancers including lung cancer^8^, breast cancer^9^, and prostate cancer^10^,^11^. In addition, studies have shown that exRNAs are functionally active. For example, the release of extracellular miRNAs is associated with anti-cancer signaling^12^ or cancer metastasis signaling^13^. miR-143 within extracellular vesicles inhibited proliferation of cancerous cells^14^ whereas introduction of miR-16 enriched extracellular vesicles into prostate cancer cells significantly suppressed expression of miR-16 target genes^15^. These studies suggest that exRNAs not only have important function in cell-cell communications but may also serve as attractive candidates for disease biomarkers.

Currently, biomarker studies using extracellular vesicles are faced with at least two great challenges. The first is a lack of systematic evaluation of the technical effect of RNA-seq library preparation on RNA abundance and detectability. The second is a lack of large scale RNA-seq data in a variety of populations including both healthy individuals and those with diseases to generate exRNA expression profiles as a baseline reference. Previously, we have examined three plasma samples by RNA-seq, evaluated technical variations among different RNA-seq library protocols and presented plasma exRNA composition^16^. Recently, a new study compared the landscape of small RNAs in human saliva across different cell types and body fluids^17^. However, these prior studies are relatively small and are not able to serve as reference exRNA profiles. To thoroughly examine the exRNA composition and distribution in plasma, we applied our extracellular vesicle isolation and RNA sequencing pipeline^16^,^18^, and examined the RNA landscapes from 192 individuals with various age, sex and health conditions. We identified sources of significant technical bias during RNA-seq library preparation and presented candidate RNA transcripts showing association with age, sex and type of cancers.

## Results

### RNA-seq data distribution

**Figure 1:**
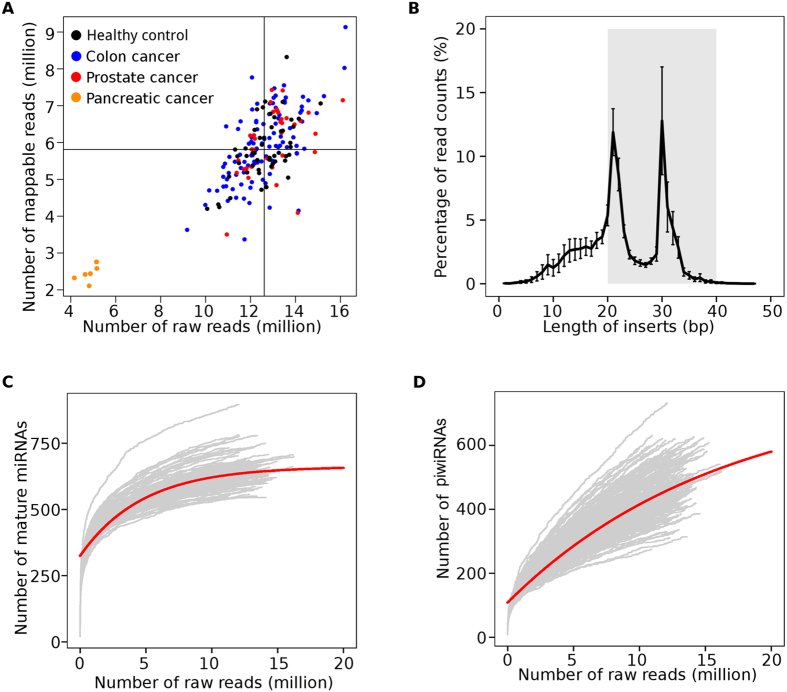
Quality control of sequencing libraries. (**A**) Number of raw reads vs. number of mapped reads. The horizontal and vertical lines were the median levels of raw reads and mapped reads, respectively. (**B**) Size distribution of sequencing library inserts. The theoretical gel-size selection region is shown in grey. (**C**) Mature miRNA saturation analysis using raw read counts. (**D**) piwiRNA saturation analysis using raw read counts. The solid red lines in (**C,D**) were modeled by non-linear regression.

To generate an exRNA profile derived from plasma extracellular vesicles, we examined 192 subjects with various health conditions (Table 1). We first evaluated the 192 RNA-seq libraries for the abundance of uniquely mapped reads, size distribution of RNA inserts, and depth of sequencing. The raw sequencing data have been deposited in the GEO database (accession number: GSE71008). On average, we achieved 12.5 million raw reads (ranging from 4 to 16 million) (Fig. 1A). Of these raw reads, 30–65% was uniquely mapped to the reference RNA sequences. Among the mapped RNAs, we examined size distribution of library inserts and found that most inserts were between 20 and 40nt with peaks at 21nt and 29nt (Fig. 1B). The two peak sizes were attributable to the top abundant small RNAs (miRNAs and piwiRNAs). To determine if sequencing depth had any effect on the RNA detectability, we examined the association between raw read number and uniquely mapped read numbers. We found that the number of unique miRNAs plateaued at 10 million raw reads (Fig. 1C). The number of detectable unique piwiRNAs, however, kept significant increase even after 15 million raw reads (Fig. 1D).

**Figure 2:**
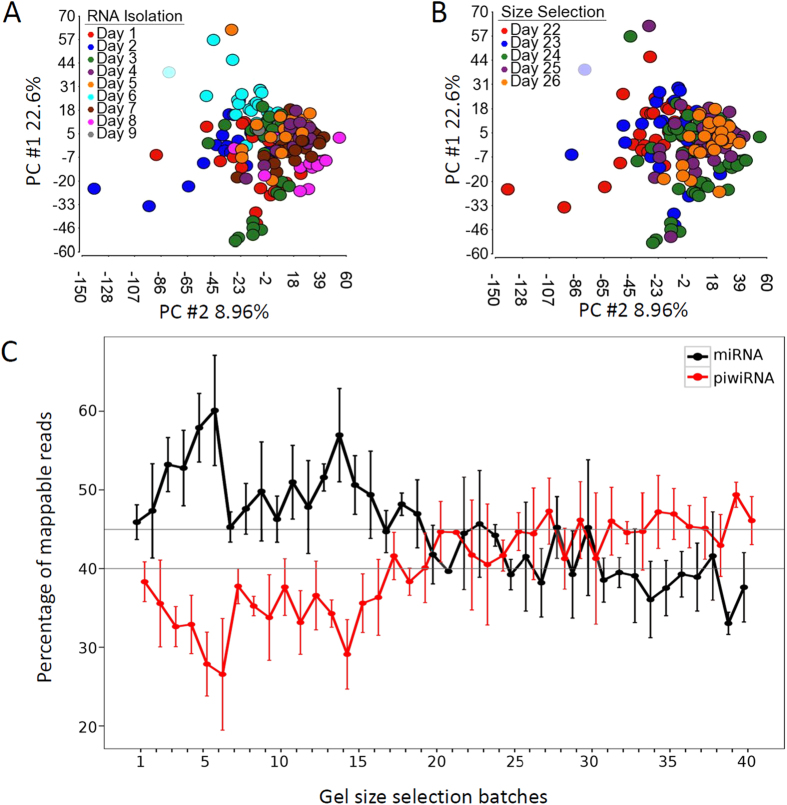
Technical effects on RNA expression profiles. (**A**) Principal component analysis showing effect of RNA isolation dates on RNA profile changes. (**B**) Principal component analysis showing effect of gel size selection dates on RNA profile changes. (**C**) Effect of gel size selection on the percentage distribution of miRNAs (precursor and mature miRNAs) and piwiRNAs. Batches of 40 polyacrylamide gels are shown. The upper and lower horizontal lines are the median levels of miRNAs and piwiRNAs, respectively.

To test if technical factors had any effect on distribution of detectable RNAs, we performed principal component analysis and observed significant bias of RNA extraction and gel size selection dates (Fig. 2A,B). To examine if the bias affected distribution of any specific RNA species, we grouped the 192 samples based on gel size selection dates and examined percentage changes of RNA species among different samples. We found that the miRNA components were significantly higher in the first 19 gels than in the remaining 21 gels (Fig. 2C). This analysis demonstrated clear batch effect that caused systematic variations of RNA abundant levels and distribution of RNA species.

### Diversity of exRNA species

**Figure 3:**
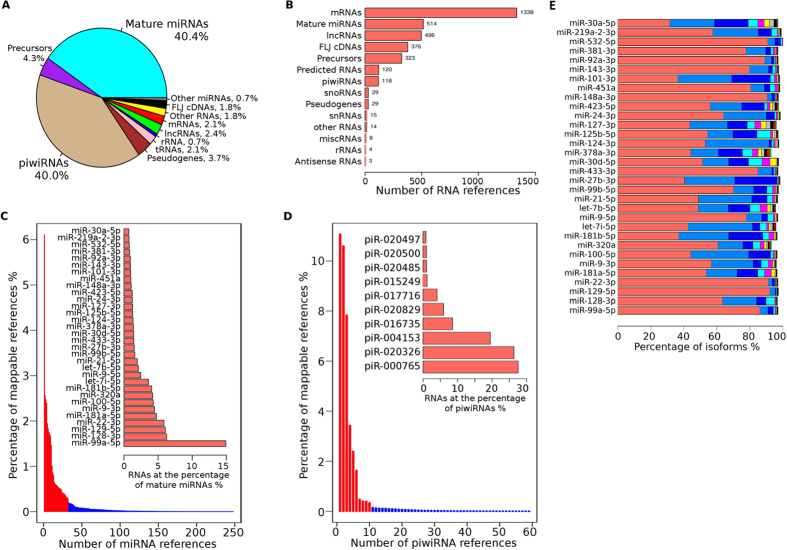
Statistical summary of RNA species detected by RNA-seq across 192 libraries. (**A**) Percentage of each RNA species in all mapped RNAs. (**B**) Number of mapped unique RNA references in different RNA species. (**C**) Percentage (sorted from high to low) of each detected mature miRNAs in all mapped miRNA reads. The top 32 miRNAs (quantile 0.98) are highlighted in red and are also shown in embedded graph. (**D**) Percentage (sorted from high to low) of each detected piwiRNAs in all mapped reads. Top 10 piwiRNAs (quantile 0.98) are highlighted in red and also shown in embedded graph. RNA transcripts with >4 RPM were used. (**E**) The top 32 miRNAs and their isoform proportions. The color bars represent the percentage of each miRNA isoform.

To examine the diversity of exRNA species, we applied an iterative strategy by mapping each of the RNA reference databases in sequential order. This analysis showed that miRNA and piwiRNA were the most abundant RNA species (Fig. 3). Of those uniquely mapped reads, approximately 40.4% of reads were mapped to 514 mature miRNAs, and 40% of reads were mapped to 118 piwiRNAs. We found that the 32 most expressed miRNAs (0.98 quantile) accounted for 86.5% of all detected miRNAs and the 10 top piwiRNAs were attributable to 96% of all detected piwiRNAs. Not surprisingly, among all RNA species, the top three most abundant RNAs were piwiRNAs including piR_000765, piR_020326, and piR_004153 while the most abundant miRNA, miR-99a-5p, ranked 4th. Meanwhile, we identified 1,338 unique mRNAs (2.1%), 496 lncRNAs (2.4%), 29 snoRNAs (3.7%), 29 pseudo genes (3.7%) and other annotated RNAs (predicted RNAs and FLJ cDNAs). The most noticeable mRNAs included C15orf52, ST8SIA1, and MTRNR2L5. Top lncRNAs were C1orf213, LINC00324, and LOC388692. Additionally, we also observed nonhuman miRNA species. The most abundant was a cattle miRNA bta-miR-6529a, which ranked as the 39^th^ most abundant RNA transcript with average log2 expression value being 11.89 (range from 11.12 to 12.79). Supplementary Table S1 shows our complete list of the 493 RNA transcripts with log2 transformed RPM >5.

Like mRNA transcripts, miRNAs also demonstrate variant transcripts (isoforms), characterized by slightly different sizes and nucleic acid positions. To evaluate miRNA isoform distribution, we categorized each mapped miRNA based on its length and position. We found isoforms in all commonly detected miRNAs. For example, the top 32 abundant miRNAs demonstrated a wide variety of isoforms (Fig. 3E). Some miRNAs such as miR-30a-5p, 101-3p, 27b-3p, and 181b-5p showed several major isoforms while others such as miR-532-5p, 92a-3p, 148a-3p, 22-3p and 129-5p showed only one dominant isoform. A complete list of these miRNA isoforms is provided in the supplementary file (Supplementary Table S2).

### Stably expressed exRNAs

**Figure 4:**
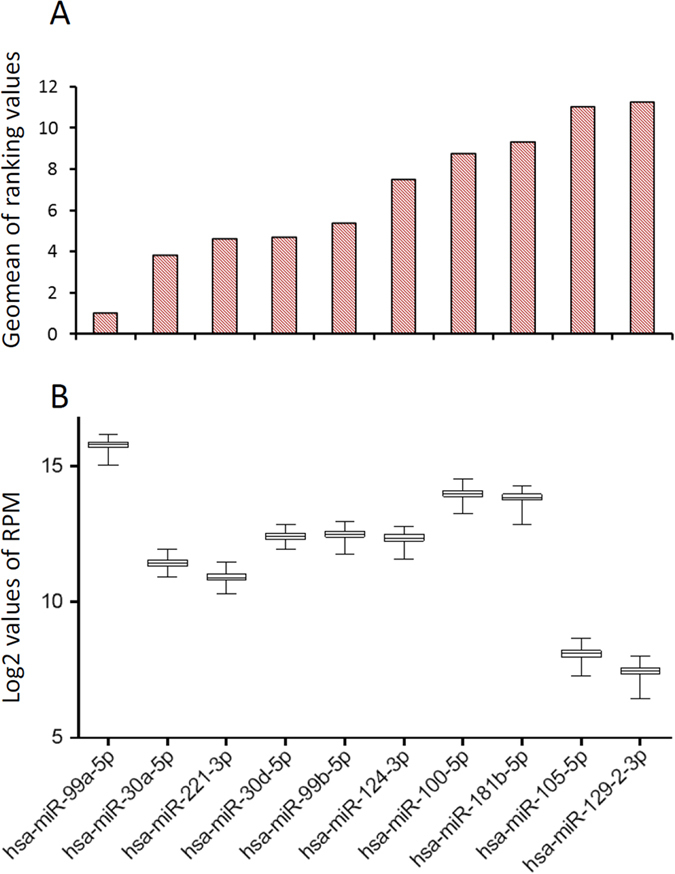
exRNA stability testing. (**A**) Ten miRNAs with the highest stability. Smaller ranking values had higher stability. (**B**) Boxplot showing abundance level of each corresponding miRNA.

To date, there have been no well-established RNAs to serve as a reference baseline for quantitative analysis of target exRNAs. To identify such reference RNAs, we first selected 60 RNAs with the smallest coefficient of variance (CV). Among those selected, 48 were from miRNAs. We applied RefFinder to identify the most stably expressed RNAs. This analysis revealed various expression stabilities among 60 selected transcripts across the 192 samples (Supplementary Table S3). Because lower ranking values generally indicated better expression stability, based on their geometric means, we found that all of the top ten most stably expressed RNAs were from miRNA species (Fig. 4). Among those, miR-99a-5p, miR-30a-5p and miR-221-3p were the top three stably expressed RNA transcripts. In contrast, another small RNA species, piwiRNA, was among the least stably expressed RNAs. The best piwiRNA (piR_015249) was ranked 47^th^ among the 60 selected transcripts.

### Age- and sex-associated exRNAs

**Figure 5:**
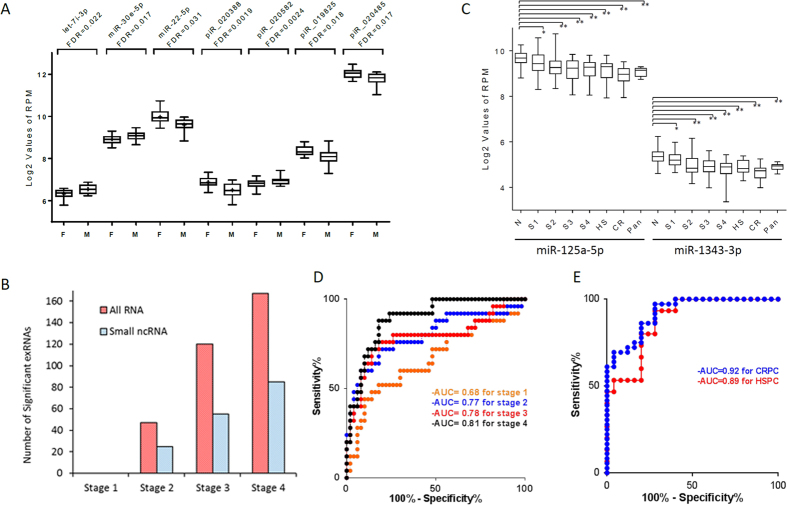
Association of exRNAs with biological and clinical characteristics. (**A**) Boxplot of sex-associated small RNAs in 50 healthy samples. (**B**) Stage-dependent analysis of differentially expressed RNA transcripts in colon cancer. Number of colon cancer-associated RNA transcripts increased with disease progression. (**C**) Association of selected miRNAs with cancer types and clinical stages. Abundance levels of miR-125a-5p and miR-1343-3p were constantly lower in all cancer types and stages. S1-4: stages I–IV. HS: hormone sensitive prostate cancer. CR: castration resistant prostate cancer. Pan: pancreatic cancer. *FDR > 0.05. **FDR < 0.05. (**D**) Colon cancer statistical model showing AUC values from 0.68 to 0.81 in clinical stages I–IV. (**E**) Prostate cancer model demonstrating higher AUC values from 0.89 in HSPC to 0.92 in CRPC, likely contributed by more advanced stages in prostate cancer patients.

To estimate age and sex effect on exRNA abundance, we first excluded all cancer patients from this analysis due to potential influence of disease status. We then performed analysis of covariance (ANCOVA) in 50 healthy controls by adjusting experimental factors (RNA isolation and gel size selection dates) and biological factors (age or sex). This analysis revealed 20 exRNA transcripts to be significantly associated with sex and 15 RNA transcripts with age (FDR < 0.05) (Supplementary Table S4). Among the 15 age-associated RNAs, seven were protein-coding genes, three were lncRNAs and five were other long transcripts. In contrast, the 20 sex-associated RNA tended to be short ncRNAs. Among those were seven small ncRNAs, 11 tRNAs, one lncRNA, and one other transcript. These sex-associated small ncRNAs included four piwiRNAs (piR_020388, piR_020582, and piR_020485, piR_019825) and three miRNAs (miR-30e-5p, let-7i-3p and miR-22-5p) (Fig. 5A).

### Small RNA signatures in cancer patients

To identify an exRNA signature for colon cancer, we applied ANCOVA by including disease status, age, sex, RNA isolation and gel size selection to examine expression differences between 100 colon cancer patients and 50 healthy controls. We compared each stage (I–IV, N = 25 per stage) to the controls separately. This analysis identified 1, 47, 120, and 167 unique RNA transcripts showing significant differences (FDR < 0.05) in stage I, II, III, and IV, respectively. Among these, small ncRNAs (mature miRNAs and piwiRNAs) were the most common RNA species, accounting for 0, 25, 55, and 85 individual RNAs for stages I–IV (Table 2). Clearly, the number of RNA transcripts with significant changes increased with disease progression (Fig. 5B). We also performed the same analysis to identify RNA transcripts showing abundant level differences between prostate cancer and male controls, and between pancreatic cancer and controls (Table 2). Of all significant changes, two miRNAs (miR-125a-5p and miR-1343-3p) were significantly decreased in all evaluated types of cancer (Fig. 5C). The statistical significance for all RNA transcripts is listed in Supplementary Table S5. It is worth mentioning that some other RNA species also showed significant differences. For example, the transcripts C6orf226 (NM_001008739), C-CAS09594 (FLJ cDNA) and LOC388692 (lncRNA) were significantly different between colon cancer stages II–IV and healthy controls. *CDHR1* and *PAQR5* were the most significantly differential transcripts in prostate cancer and pancreatic cancer patients, respectively.

To evaluate if the significant small ncRNAs were able to differentiate cancer patients from healthy controls, we first performed 10 fold cross-validation analyses on miR-125a-5p and miR-1343-3p, two most commonly down-regulated miRNAs in the dataset, for their ability of cancer classification. We found that miR-1343-3p alone was able to generate an area under the curve (AUC) of 0.59, 0.72, 0.73, 0.82 for colon cancer stages I, II, III and IV, 0.75 and 0.87 for HSPC and CRPC, respectively. miR-125a-5p alone was able to generate an AUC of 0.62, 0.76, 0.74, 0.77 for colon cancer stages I, II, III and IV, 0.77 and 0.86 for HSPC and CRPC, respectively. We then performed stepwise selection using 60 small ncRNAs (colon cancer) and 46 small ncRNAs (prostate cancer) with FDR < 0.05 (based on Supplementary Table S5). We identified six (colon cancer) and seven (prostate cancer) small ncRNAs that contributed independently to discriminate patients from controls. These small transcripts included miR-1343-3p, miR-125a-5p, miR-708-5p, miR-381-3p, miR-543 and piR_019825 for colon cancer, and miR-125a-5p, miR-1343-3p, let-7d-5p, miR-191-5p, miR-204-5p, miR-7-5p and piR_016658 for prostate cancer. The six exRNA multivariate model predicted colon cancer with an AUC of 0.68, 0.77, 0.78, and 0.81 for stages I, II, II, and IV, respectively (Fig. 5D). Clearly, prediction accuracy increased with colon cancer stage advancement. The seven exRNA model was able to classify prostate cancer with an AUC of 0.89 and 0.92 for HSPC and CRPC, respectively (Fig. 5E). Overall, these multivariate models showed an AUC of 0.76 for all colon cancer patients (sensitivity = 0.72 and specificity = 0.80) and 0.91 for all advanced prostate cancer patients (sensitivity = 0.94 and specificity = 0.88). Detail predictive characteristics including sensitivity, specificity, positive/negative predictive value and AUC were shown in Supplementary Table S6.

## Discussion

In this study, we applied RNA-seq to examine plasma exRNA profiles from 192 individuals and evaluated a variety of RNA species for their abundance, stability and age/gender correlation as well as disease association. Studies have shown that rRNAs are the most common species when preparing RNA-seq libraries with 15–70 nt inserts^19^ or without insert size selection^20^. We previously performed gel size selection of 20–40nt inserts during library preparation and observed significant increase of miRNA content^16^. The study also tested different library preparation kits and found the significant effects of sequencing preparation methods on miRNA abundance. By systematic analysis of the 192 subjects, this new RNA-seq data have further shown that the experimental procedures can cause constitutive variations in RNA abundance and result in a significant batch effect among the subjects tested. Therefore, exRNA profiles could vary significantly if using different methods for vesicle isolation, RNA extraction, sequencing library preparation or gel size selection. We highly recommend consideration of the technical variations during statistical analysis and sample size power calculation. Due to small fold changes among disease groups, larger sample size is clearly needed. Additionally, it is critically important to standardize the exRNA analytical procedure from exRNA isolation to library preparation.

By far, qRT-PCR is the most commonly used approach for gene expression quantification. However, to quantify candidate RNA abundances, it is essential to normalize their expression levels to well established reference controls. Unfortunately these controls have not yet been established for exRNAs^21^,^22^. It has been reported that exRNAs in circulating vesicles are selectively enriched from parent cells^23^, therefore, endogenous RNA controls for cellular RNA normalization are not suitable for exRNA normalization. To normalize qRT-PCR results, previous studies have used spike-in controls or miR-16-5p as reference controls. However, exogenous spike-in miRNAs such as *Caenorhabditis elegans* miR-39/54/238 barely correct for variations of RNA extraction efficiency and do not have the capacity of normalizing variations under different biological and pathological conditions. The use of miR-16-5p as an internal control has been questioned because red blood cells are an important source of miR-16-5p^22^,^24^. In fact, miR-16-5p levels may increase by 20–30 fold in serum/plasma prepared from hemolyzed specimens^25^. In this study, we report a systematic survey of exRNA profiles in the largest population to date representing both genders and individuals with diverse ages and health conditions. Based on the data, miR-99a-5p clearly demonstrates the highest abundance and stability among all exRNAs examined. Other best reference candidates include miR-30a-5p, miR-221-3p and miR-30d-5p. However, further confirmation is needed to use these reference candidates for exRNA quantification.

It is well known that miRNAs are the most abundant circulating extracellular small RNAs in both healthy controls and cancer patients. However, our extracellular RNA-seq data show that another small RNA species, piwiRNA, is almost equally as abundant as miRNA in plasma extracellular vesicles. piwiRNAs are 25–33nt in length and lack sequence conservation^26^,^27^. Our saturation analysis shows that a sequencing depth of 10 million raw reads is sufficient for miRNA quantification analysis. For piwiRNAs, however, higher sequencing depth is needed to fully decipher diversity and distribution, which may be caused by the huge number of piwiRNAs in human genome. Based on piRNABank (http://pirnabank.ibab.ac.in/stats.html), there are a total of 32194 unique piwiRNAs, which is over 10 fold more than annotated miRNAs in human. It has been reported that the biogenesis of piwiRNAs is independent of Dicer and requires other nucleases^28^. To date, the roles of piwiRNA are yet to be being fully explored^29^. Most functional studies on piwiRNA are mainly carried out in model organisms such as *C. elegans*, *Drosophila*, zebrafish, mice and rats^30^. These studies have illustrated the multifaceted somatic functions of the pathway not only in transposon silencing but also in genome rearrangement and epigenetic programming with biological roles in stem-cell function, whole-body regeneration, memory and possibly cancer^27^.

In addition to the small ncRNAs (miRNAs and piwiRNA), we also detected other types of RNAs and nonhuman miRNAs. The most common mRNA transcript is a protein coding gene *C15orf52* with an important paralog being *CCDC9*. The second most common mRNA is *ST8SIA1*, which encodes a type II membrane protein that catalyzes the transfer of sialic acid from CMP-sialic acid to GM3 to produce gangliosides GD3 and GT3. This protein may be found in the Golgi apparatus and is a member of glycosyltransferase family 29. Ganglioside GD3 is known to be important for cell adhesion and growth of cultured malignant cells^31^. The third most common mRNA is *MTRNR2L5*, a Humanin-like protein that has shown strong cytoprotective actions against various stress and disease models^32^. Additionally, we consistently detected nonhuman miRNAs, some of which showed extremely high expression levels across all 192 human plasma samples. An example is the highly abundant bta-miR-6529a, a cattle miRNA without any known miRNA homolog in human miRbase. Although the 21nt sequence can be perfectly mapped to a unique human genome position at chr3:119803462-119803482, there is no clear indication that this region is transcriptionally active. Whether this exogenous miRNA is derived from human food consumption, how it gets into blood stream and what is its effect on human health remain unclear.

Our study shows that both sex and age are significant contributors to variations observed in abundance of some exRNA levels. The most significant association with age was found in three protein coding genes (*CSF2RA*, *DLD*, and *RRP1B*). *CSF2RA* is the alpha subunit of the heterodimeric receptor for colony stimulating factor, a cytokine that controls the production, differentiation, and function of granulocytes and macrophages. *DLD* is a component of the glycine cleavage system and the alpha-ketoacid dehydrogenase complexes. This gene is involved in the hyperactivation of spermatazoa during capacitation and in the spermatazoal acrosome reaction. *RRP1B* is a nucleolar protein that regulates E2F1-induced apoptosis^33^ and plays a role in the rapid transduction of cellular signals that call for regulation of ribosome production in response to cellular stress and/or changes in growth conditions^34^. In addition, we found significant association with sex in three miRNAs including miR-30e-5p, let-7i-3p, and miR-22-5p. Interestingly, miR-30e-5p is relatively stable and has been successfully used as reference control in our previous prostate cancer (male only) survival study^11^. let-7i-3p and miR-22-5p have shown association with various disease conditions^35^,^36^,^37^. These results demonstrate that biological and pathological conditions have significant effect on some exRNA abundances.

Our study also shows that changes of RNA abundance in plasma extracellular vesicles are cancer-related. In particular, two miRNAs (miR-125a-5p and miR-1343-3p) were constantly downregulated in colon, prostate and pancreatic cancers including early stage colon cancer. Although regulatory mechanism is not clear, lower expression in tumor tissues than normal control tissues in previous studies^38^,^39^,^40^,^41^ suggest their tumor-originated downregulation. miR-125a-5p has been shown to inhibit cell proliferation and promote cell differentiation by targeting critical genes^38^,^40^,^42^,^43^, and has been reported as a potential diagnostic and prognostic biomarker for a variety of cancers^38^,^39^,^40^,^41^,^42^. For miR-1343-3p, however, little is known about its functional role or specific disease association. The only report shows that the miR-1343-3p is one of 4 miRNAs that differentiate pancreato –biliary malignant from non-malignant diseases^44^. Additionally, based on the differentially expressed exRNAs, we have also developed multivariate models for diagnostic classification. These diagnostic models show a clear trend of improved prediction power with more advanced cancer stage. If further validated, due to noninvasive and easily accessible, plasma exRNAs may serve as ideal biomarker candidates for disease diagnosis. To fully realize the clinical application, future study is needed to develop a multiplex-based and highly sensitive exRNA quantification assay. Future study is also needed to test diagnostic specificity of the statistical models in patients with various cancers and disease conditions. It will be more informative if these studies also include matched tumor tissues to decipher origin of exRNA biomarkers. Regardless, the current dataset has provided a rich resource of exRNA profiles across a large human population, paving the way for clinical evaluation of the exRNAs for their disease association.

In conclusion, we examined exRNA profiles in plasma samples derived from 192 individuals. This is so far the largest study to profile RNA contents of these circulating vesicles by RNA-seq in healthy and diseased individuals. Our data show that RNA-seq library preparation procedures can introduce significant technical bias in the detection of different RNA species and abundance of individual RNAs. This result highlights the importance of establishing standardized techniques, protocols, and workflows for extracellular vesicle isolation and RNA sequencing library preparation. We also identified a set of RNAs (in particular, miRNAs) with relatively stable expression which may serve as reference candidates for exRNA quantification. Additionally, we identified a panel of RNA transcripts in plasma extracellular vesicles showing association with age, sex, and cancers. Although further study is needed to validate our findings, this study has clearly expanded our knowledge of extracellular vesicle biology and exRNAs.

## Methods

### Study subjects and plasma preparation

Peripheral blood samples were collected from a total of 192 subjects including 50 healthy controls with a median age of 54 (ranging from 25 to 79), 100 colon cancer patients with a median age of 55 (ranging from 15 to 90) (N = 25 for each of stages I–IV), 36 prostate cancer patients with a median age of 69 (ranging from 49 to 87) including 21 castration-resistant and 15 hormone-sensitive patients, and 6 pancreatic cancer patients with median age of 69 (ranging from 59 to 80) (Table 1). For the 36 prostate cancer patients, 23 were used for case only survival analysis in our previous publication^11^. With the exception of the prostate cancer patients who were men only, the gender distribution was equal in healthy controls, colon cancer and pancreatic cancer patients. Ages from healthy controls were matched to colon cancer patients. Peripheral blood samples from these specimens were collected in 10 ml plasma separator tubes. Within 2 hours after collection, the plasma samples were fractioned into multiple aliquots after centrifugation at 2,000 × *g* for 10 min, leaving >1 ml supernatant and cell debris intact in the original tubes. All plasma samples were stored at −80 °C until use. To avoid batch effect, we randomized the 192 subjects during RNA extraction, sequencing library preparation, gel size selection and sequencing pooling. Informed consent was obtained from all study participants prior to blood draw. This study was approved by Mayo Clinic and the Medical College of Wisconsin Institutional Review Boards. The patient recruitment and informed consent were carried out in accordance with the approved guidelines from both institutions.

### RNA isolation from extracellular vesicles

The vesicle isolation and RNA extraction have been published in our previous study^16^. In brief, plasma samples were centrifuged at 3,000 × *g* for 10 min to remove possible residual cell debris after thawing the stored samples. The supernatants were incubated with pre-warmed thromboplastin D (Thermo Fisher Scientific, Grand Island, NY) for 15 min and mixed with ExoQuick Exosome Precipitation Solution (SBI, Mountain View, CA). RNase A (Sigma, St. Louis, MO) was then added to a final concentration of 10 μg/ml and incubated at 4°C overnight to remove free RNAs in plasma. 150 units/ml of murine RNase inhibitor (NEB, Ipswich, MA) was added followed by precipitating the extracellular vesicles by centrifugation at 1,500 × *g* for 30 min. The vesicle pellets were dissolved in 25μL of PBS and RNA was extracted immediately. RNA was isolated with miRNeasy Micro Kit (QIAGEN, Valencia, CA) following the standard manufacturer protocol. To eliminate potential co-precipitated DNA, the column-bound RNA was treated with 30 units of DNase I for 15 min at room temperature. The RNA was then eluted with 14μl of DNase- and RNase-free water. RNA quality and quantity were estimated by Agilent Bioanalyzer 2100 using a Small RNA Chip (Agilent, Santa Clara, CA).

### Small RNA sequencing libraries preparation

Small RNA libraries were constructed following manufacturer instructions using the NEBNext Multiplex Small RNA Library Prep Set for Illumina (NEB) with slight modifications. 2-10ng of RNA per sample were reversed transcribed into cDNA after ligation of the multiplex 3′ SR Adaptor, hybridization of the reverse transcription primer, and ligation of the multiplex 5′ SR Adaptor. RNA libraries were amplified by 10 PCR cycles using Illumina compatible index primers. 12 sequencing libraries were pooled into a single sequencing lane. The amplified libraries were resolved on a native 5% polyacrylamide gel (Bio-Rad, Hercules, CA). DNA fragments corresponding to 140–160bp (small RNA inserts plus 3′ and 5′ adaptors) were recovered in 12 μl of Elution Buffer (QIAGEN). Libraries were quantified by real time qPCR using KAPA Library Quantification Kits (KAPA Biosystems, Wilmington, MA) and subjected to sequencing using an Illumina HiSeq2000 analyzer.

### RNA-seq data mapping and scaling

The RNA-seq analytical pipeline eRNA (v1.2)^18^ was developed for data analysis, including extraction of raw data, 3′ adaptor trimming, sequence alignment, and read count scaling. We applied an iterative alignment strategy: unmapped sequences from the previous alignment were used for the mapping of the next RNA reference species. The sequential alignment followed the order of mature miRNA, precursor miRNA, piwiRNA, siRNA, lncRNA, mRNA, pseudo genes, antisense RNA, rRNA, snRNA, snoRNA, misc-RNA, other transcribed RNA, FLJ human cDNA, and predicted RNA. The references for human miRNA, piwiRNA, siRNA and FLJ human cDNA were downloaded from miRBase (http://www.mirbase.org, Release 21), piwiRNABank (http://piwiRNAbank.ibab.ac.in), siRNDdb (http://siRNA.cgb.ki.se), and FLJ Human cDNA Database (http://flj.lifesciencedb.jp/top/, v3.2). The other human genome references were downloaded from NCBI (ftp://ftp.ncbi.nlm.nih.gov.genomes/Homo_sapiens/, Release 106). The aligner Bowtie (version 1.1.1) was integrated into eRNA, where the parameters for sequencing alignment were −l 18, −v 1, −m 2, and –norc –best –strata. To make the sequencing profiles comparable, we scaled (normalized) RNA profiles as read count of a target RNA per million mapped reads (RPM).

### miRNA isoform analysis

For each miRNA, aligned sequences were grouped by nucleotide sequence length and position. Only sequences with more than 1920 combined read counts (i.e. 10 read counts/sample) across all individuals were analyzed and annotated with a numeric suffix to identify the isoform. For example, the most common nucleotide sequence aligning to hsa-let-7d was designated hsa-let-7d.1, the second most common as hsa-let-7d.2, followed by hsa-let-7d.3, and so on. Nucleotide sequences not meeting the 1920 read criterion were all grouped together for each miRNA and were annotated by the “pool” isoform suffix (e.g. hsa-let-7d.pool).

### exRNA stability test

We performed RNA stability testing using Ref Finder to determine the most likely reference candidates for normalization (http://www.leonxie.com/referencegene.php?type=reference). RefFinder was developed for evaluating and screening reference genes from experimental datasets. It integrates the currently available major computational programs (geNorm, Normfinder, BestKeeper, and the comparative ΔCt method)^45^,^46^,^47^,^48^ to compare and rank the tested candidate reference genes. Based on the rankings from each program, RefFinder assigns an appropriate weight to an individual transcript and calculates the geometric mean of their weights for the overall final ranking. The exRNAs with lower geometric mean are theoretically more stable than those with higher geometric mean.

### Statistical analysis of significant exRNAs

To accurately estimate statistical significance of each exRNA, we summarized the expression values (log 2-transformed) by their mean and standard deviation within the study groups and used analysis of covariance (ANCOVA) approaches to compare the abundance level differences between study groups for each exRNA while adjusting for experimental variables (RNA isolation and gel size selection) and biological variables (age, sex and cancer status) (Partek Genomics Suite 6.1, Partek Inc, St. Louis, MO). Because 493 exRNAs were included in the analysis, we employed a false discovery rate (FDR) approach and computed a q-value^49^ to adjust for multiple testing. RNA transcripts with FDR of less than 0.05 were considered to be significant.

### Multivariate models for cancer prediction

To build multivariate models for differentiation between cancer patients and healthy controls, we first selected exRNA candidates that showed differential expression between cancer stages and controls (FDR < 0.05). We then performed multivariate feature selection with optimal feature number from 3 to 10 exRNA candidates. 10 fold cross-validation was included during the feature selection process. The classification methods were k-nearest neighbor, discriminant analysis, support vector machine and logistic regression. For each model, the AUC value along with sensitivity, specificity, positive predictive value, and negative predictive value was automatically reported (Partek Genomics Suite). This AUC value, often referred to as the c-statistic, examines all possible case control pairs and measures the proportion of the time the statistical model predicts higher risk for the case^50^.

## Additional Information

**How to cite this article**: Yuan, T. *et al.* Plasma extracellular RNA profiles in healthy and cancer patients. *Sci. Rep.* **6**, 19413; doi: 10.1038/srep19413 (2016).

## Supplementary Material

Supplementary Table S1

Supplementary Table S2

Supplementary Table S3

Supplementary Table S4

Supplementary Table S5

Supplementary Table S6

## Figures and Tables

**Table 1:** Clinical characteristics of 192 subjects.

	Sex		Age		Notes
	M	F	Average	Range	
Healthy Controls	25	25	54	25–79	
Colorectal Cancer	50	50	55	15–90	25 cases for each of stages I–IV
Prostate Cancer	36	NA	69	49–87	HSPC = 15, CRPC = 21
Pancreatic Cancer	3	3	70	59–80	2 cases for each of borderline, resectable and metastatic stages

HSPC-hormone sensitive prostate cancer, CRPC-castration-resistant prostate cancer.

**Table 2:** Association of small exRNAs with human cancers.

Gene Name	RNA Categories	False Discovery Rate (FDR)			
Colorectal Cancer		Stage I (25)	Stage II (25)	Stage III (25)	Stage IV (25)
hsa-miR-1343-3p	Mature miRNA	2.35E-01	8.52E-03	5.48E-04	1.45E-05
hsa-miR-125a-5p	Mature miRNA	1.85E-01	9.03E-03	5.48E-04	2.05E-05
hsa-miR-381-3p	Mature miRNA	1.85E-01	3.33E-02	9.57E-04	2.05E-05
hsa-miR-543	Mature miRNA	1.85E-01	1.74E-02	3.51E-03	3.53E-05
hsa-miR-128-3p	Mature miRNA	1.80E-01	1.98E-02	1.74E-02	6.67E-05
hsa-miR-139-5p	Mature miRNA	1.80E-01	2.82E-02	1.05E-02	2.66E-04
hsa-miR-212-5p	Mature miRNA	2.49E-01	4.21E-02	2.05E-02	4.04E-04
hsa-miR-92b-3p	Mature miRNA	1.80E-01	4.21E-02	1.16E-02	7.69E-04
hsa-miR-708-5p	Mature miRNA	4.01E-01	1.74E-02	1.00E-02	1.33E-03
hsa-miR-132-3p	Mature miRNA	3.28E-01	4.21E-02	3.16E-03	6.19E-03
hsa-miR-885-5p	Mature miRNA	1.98E-01	3.03E-02	1.54E-02	1.10E-02
has-piR-019825	piwiRNA	4.53E-01	3.45E-03	2.25E-02	2.13E-02
Prostate Cancer		HSPC (15)		CRPC (21)	
hsa-miR-1343-3p	Mature miRNA	3.09E-05		9.76E-05	
hsa-miR-125a-5p	Mature miRNA	1.22E-03		3.74E-04	
has-piR-016658	piwiRNA	2.06E-04		4.06E-04	
hsa-miR-146a-5p	Mature miRNA	2.30E-04		5.59E-04	
hsa-miR-219a-5p	Mature miRNA	7.80E-04		8.66E-04	
has-piR-020496	piwiRNA	2.25E-04		9.03E-04	
Pancreatic Cancer		All stages (6)			
has-piR-001311	piwiRNA	8.81E-05			
hsa-miR-7-5p	Mature miRNA	6.70E-03			
hsa-miR-125a-5p	Mature miRNA	9.28E-03			
hsa-miR-1343-3p	Mature miRNA	1.90E-02			
has-piR-016658	piwiRNA	4.49E-02			
